# Shelf life estimation of Blackberry (*Rubus glaucus* Benth) with bacterial cellulose film coating from *Komagataeibacter xylinus*

**DOI:** 10.1002/fsn3.1525

**Published:** 2020-03-17

**Authors:** Jonatan A. Toscano Ávila, David A. Terán, Alexis Debut, Karla Vizuete, Josefina Martínez, Liliana A. Cerda‐Mejía

**Affiliations:** ^1^ Faculty of Sciences and Food Engineering and Biotechnology Universidad Técnica de Ambato Ambato Ecuador; ^2^ Department of Research and Development Universidad Técnica de Ambato Ambato Ecuador; ^3^ Institute for Applied Sustainability Research Quito Ecuador; ^4^ Centro de Nanociencia y Nanotecnología Universidad de las Fuerzas Armadas ESPE Sangolquí Ecuador; ^5^ Faculty of Biology Department of Genetics, Microbiology and Statistics Universitat de Barcelona Barcelona Spain

**Keywords:** bacterial cellulose, biofilm, Castile blackberry, coating

## Abstract

The Castile blackberry (*Rubus glaucus* Benth) is an Andean crop with nutritional and antioxidant properties. The intake of this fruit potentiates the immune system and reduces the risk of developing degenerative and cardiovascular diseases. However, the Castile blackberry is one of the most perishable fruits due to its high respiration rate and the lack of protectant peel, making this fruit susceptible to microbial attack and rapid deterioration. The objective of this research was to estimate the shelf life of Castile blackberry (*R. glaucus* Benth) with bacterial cellulose coating from *Komagataeibacter xylinus*, in order to improve the physicochemical and nutritional characteristics. Blackberries with bacterial cellulose coating at 4°C have extended its shelf life to 9 days and preserved the initial characteristics of texture, color, smell, and taste.

## Graphical abstract

The protection of Castille blackberry with bacterial cellulose can extent the shelf life of the fruit, leading in a better way to store it in ultrafreezing storage.

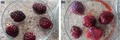

## 1 INTRODUCTION

Castile blackberry is a fruit that is appreciated for all kind of consumers, and also it is appreciated by agro‐industry and specially by the fresh producer market (Garzón, Riedl, & Schwartz, 2009). However, bad condition in pre‐ and postharvest processes, such as handling, transportation, and packaging, affects the morphological characteristics of the blackberry, decreasing the content of bioactive compounds due to the increment in soften and physical deterioration of the fruit (Bernal‐Roa, Melo, & Díaz‐Moreno, 2011). Thus, these factors increase the susceptibility of blackberry to microbial attack and rapid deterioration, which represent a problem in agricultural business.

Moreover, the demand for good quality fruits and the importance of fruit intake in the prevention of chronic diseases are the base for several studies related to improvement of procedures related to the handling and industrialization of fruits in their natural form (Pem & Jeewon, 2015). These procedures are the lead to maintain the physical, nutritional, and sensorial qualities of fruits, minimizing postharvest losses and foodborne diseases (James & Zikankuba, 2017). Currently, the continuous use of synthetic chemical preservatives in food industries generates risks for human health; therefore, it is necessary to look for a new alternative, in order to keep food safety and comply with local and international safety requirements (Li et al., 2014).

In addition, the growing demand for fresh, quality, and extended shelf life foods has encouraged the research and development of edible coatings (Tharanathan, 2003). One of the possible alternatives is the implementation of comestible coatings that does not affect the physicochemical properties of the fruit. Edible coatings have become a very promising technology to increase the postharvest conservation of fruits (Okcu, Yavuz, & Kerse, 2018). They are thin matrices produced mainly from biopolymers including polysaccharides, lipids, proteins, or a mixture of these, which are applied to the surface of the product (Quintero, Falguera, & Muñoz, 2010). These edible coatings must have mechanical properties that guarantee adhesiveness and can prevent dehydration and deterioration, but most importantly, they should not affect the color and smell of the food (Figueroa, Salcedo, Aguas, Olivero, & Narvaez, 2011).

The application of edible coatings based on polymers and natural antimicrobials could protect the fruit during storage period, increasing the strength and texture of the fruit, and maintain the overall appearance of the fruit (Méndez, 2008). Furthermore, the combination of coating and an appropriate storage temperature could prolong the postharvest life of fruits, while maintaining their sensory and nutritional quality and controlling weight lost (Almenar, Samsudin, Auras, & Harte, 2010).

In this regard, a wide variety of molecules has been used in the production of edible films and coatings (Dhumal & Sarkar, 2018). Since bio cellulose has unique properties such as biodegradability, biocompatibility, water retaining capacity, and tensile strength, bio cellulose is becoming one of the most promising material used in food packaging applications (Costa, Almeida, Vinhas, & Sarubbo, 2017).

Therefore, the aim of this study was the determination of the effect of the bacterial cellulose as coating in the shelf life of Castile blackberry.

## 2 MATERIAL AND METHODS

### 2.1 Materials

**Figure 1:**
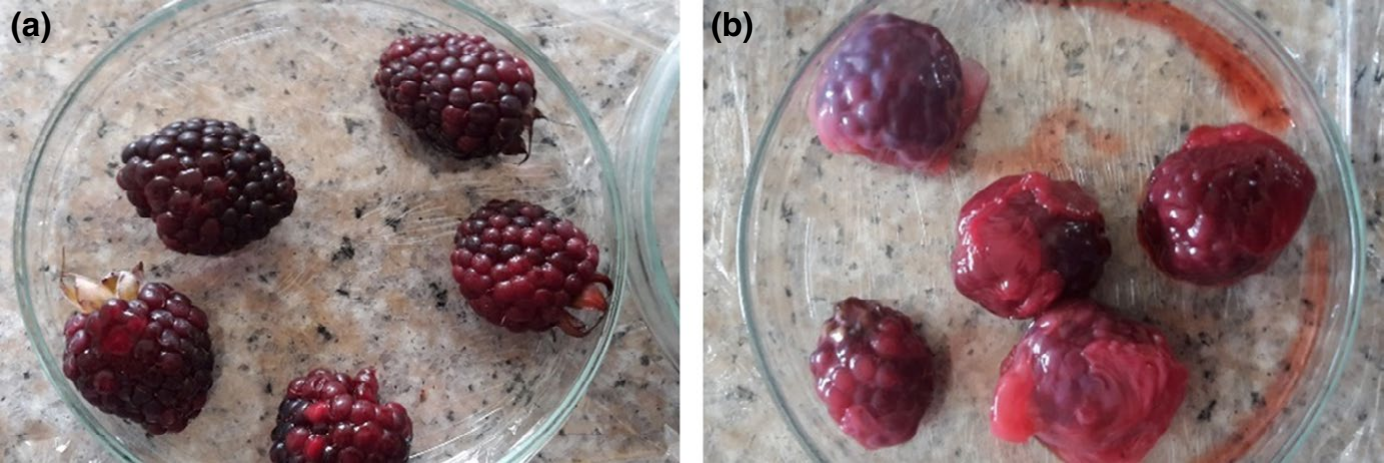
Castille blackberry with coating (a), blackberry without coating (b)

Castile blackberries (*Rubus glaucus* Benth) were collected manually in the morning from a crop located in Alobamba, Riobamba, Ecuador (Figure 1a). All berries were transported immediately to the laboratories of the Faculty of Sciences and Food Engineering and Biotechnology at the Technical University of Ambato, where all analyzes were performed. Once the berries arrived to the laboratory, a standard cleaning procedure was performed. The first cleaning treatment was with distilled water, followed with a room temperature (25°C) dry procedure. This cleaning procedure was a pretreatment for all blackberries prior experimentation.

The acetic acid bacteria (BAA) *Komagataeibacter xylinus* strain K2G30 = UMCC2756 was used to produce the bacterial cellulose. *Komagataeibacter xylinus* strain was acquired from the Deutsche Sammlung von Mikroorganismen und Zellkulturen GmbH, collected, and it was provided by Dr. Pastor's group from the Department of Genetics, Microbiology and Statistics, Faculty of Biology, Universitat de Barcelona.

### 2.2 Methods

#### 2.2.1 Bacterial cellulose production

The bacteria *K. xylinus* was used to produce the bacterial cellulose, using the methodology described by Hestrin and Schramm (1954). To produce bacterial cellulose, the standard media HS was used in petri dishes of 10 cm (Hestrin & Schramm, 1954).

Once the bacterial celluloses for coating were obtained, these films were removed from the petri dishes, washed with abundant distilled water, and then washed with NaOH (0.1 N) solution in order to clean and remove all remnant bacteria. All cleaned bacterial celluloses were dried in an oven at 60°C in order to reduce the humidity and void the effect of humidity in the results. Then, one dry bacterial cellulose was used to cover the totality of one blackberry (Figure 1b).

##### Reducing sugars analysis of the growing media for bacterial cellulose production

The concentration of reducing sugars was determined following the procedure reported by Miller (1959). The concentration of reducing sugars can be calculated by 3,5‐dinitrosalicylic acid (DNS) under certain conditions. DNS reagent was prepared with the following procedure: 0.8 g of NaOH was dissolved in 20 ml of distilled water, and then 15 g of tetrahydrated sodium, 15 g of potassium tartrate, and 0.5 g of DNS (3,5‐dinitrosalicylic acid) were added to the NaOH solution. Then, the DNS reagent was filled out to 50 ml with distilled water and stored in an amber bottle at 4°C.

The concentration of the total reducing sugars in the sample was determined using a standard curve of glucose, plotting the absorbance as a function of the concentration. To obtain this curve, solutions of 0–100 mg/ml were prepared using glucose as a standard. DNS was added to these solutions, and the absorbance was measured spectrophotometrically (ThermoFisher) at 540 nm of each concentration per sample (Ramona, Bernarda, Rómulo, & Marluy, 2012).

##### Scanning electron microscopy (SEM) of bacterial cellulose

The obtained bacterial cellulose was characterized in order to determine its microstructure. The morphological analysis of bacterial cellulose was performed in small pieces of the material with a scanning electron microscope (TESCAN Mira 3). The samples were covered with small gold particles to obtain a conductive surface for the electronic scanning.

#### 2.2.2 Castile blackberry physicochemical experiments

Two treatments per experimentation were prepared for all analysis, one with coating and a second without coating.

### 2.3 Ultrafreezing storage of blackberry (−80°C)

Samples of blackberry with coating (*n* = 18) and samples without coating (*n* = 18) were placed in a container without lid inside the ultrafreezer at −80°C ± 2 for 18 days (Figure 4a). One sample per treatment was thawed daily to perform the following analysis:

#### 2.3.1 Determination of titratable acidity (TA) of the blackberry stored at −80°C

The acidity was determined with the acid–base titration with a standardized alkali solution, expressing the titratable acidity results as the mass equivalent of citric acid. The titratable acidity determination was carried out with a standard volumetric solution of sodium hydroxide in the presence of phenolphthalein as indicator. According to Sadler and Murphy (2010), the value of correction factor for citric acid is 0.064.

The acidity calculated in this analysis was expressed as a percentage of citric acid per 100 g of fresh fruit material. Five grams of blackberry sample per treatment (with coating and without coating) were weighted on an analytical balance (Mettler Toledo) and macerated to a homogenous solution. The macerated samples were transferred to the titrator vessel of the automatic titrator (Mettler Toledo). Then, the macerated sample was filled out to 50 ml with distilled water and five drops of phenolphthalein were added to the 50 ml solution. The neutralization curve was obtained by the titration of a 0.1 N NaOH solution to the 50 ml solution. At pH = 8.13, a change in color due to of phenolphthalein occurred. When change in color occurred, titration was stopped and the volume of NaOH consumed in the titration was recorded for acidity calculation.

#### 2.3.2 Determination of total soluble solids (°Brix) of the blackberry

To measure the soluble solids of the frozen blackberry from the two treatments (with coating and without coating) stored in utrafreezing conditions after 18 days. The frozen blackberry had a progressive thawing and then was macerated with the product of the liquid formed during the thawing process. The refractive index was measured of the pulp of the blackberry at room temperature, using a refractometer (Atago).

#### 2.3.3 Maturity index

This index was calculated from the ratio between the minimum value of the total soluble solids (°Brix) and the maximum value of the titratable acidity. This index is expressed as °Brix/% citric acid following the standard (NTE INEN 2427, 2010).

#### 2.3.4 Blackberry weight reduction

The variation in weight of the samples from both treatments was recorded daily, where one blackberry per treatment was thawed and weighted per day to record the variation in weight.

### 2.4 Shelf life analysis of the blackberry

Shelf life of Castille blackberry was determined by counting molds, yeasts, psychotropic bacteria, and aerobic mesophiles at different temperatures: 4°C ± 2; 25°C ± 2, and 37°C ± 2 of the treatments (with coating and without coating). Molds and yeasts count were undertaken according to the standard (NTE INEN 1529‐10, 2013), psychotropic bacteria count was undertaken according to the standard (ICMSF, 1982), and aerobes mesophilic count was undertaken according to the standard (NTE INEN 1529‐5, 2006).

For the analysis, a hood (Optimar) was used to maintain sterile condition. Per treatment (with and without coating), a mother solution was prepared with 1 g of blackberry placed into 9 ml of peptone water in a test tube. From the mother solution, three dilutions (10^–3^, 10^–4^ and 10^–5^) per sample were prepared, and each dilution was plated in duplicate. Molds and yeasts were plated on the surface of the solid media and were incubated for 24 hr at 25°C. Psychrotrophic bacteria were plated in depth of the solid media and incubated for 24 hr at 37°C. Mesophilic aerobes were plated in the surface of the solid media and incubated for 24 hr at 37°C.

### 2.5 Statistical analysis

An AxB design with six treatments was used. Two blackberries were analyzed at different times, and the analyzes were carried out on days 1, 3, 5, and 8 in duplicate. The results were analyzed by means of an analysis of variance (ANOVA) using the R Project for Statistical Computing.

## 3 RESULTS AND DISCUSSION

The Castille blackberry for all experiments were harvested at maturity stage type 4 (dark red fruit) for the corresponding experimental analyses (Figure 1a).

### 3.1 Bacterial cellulose production

#### 3.1.1 Reducing sugars analysis of the growing media

**Figure 2:**
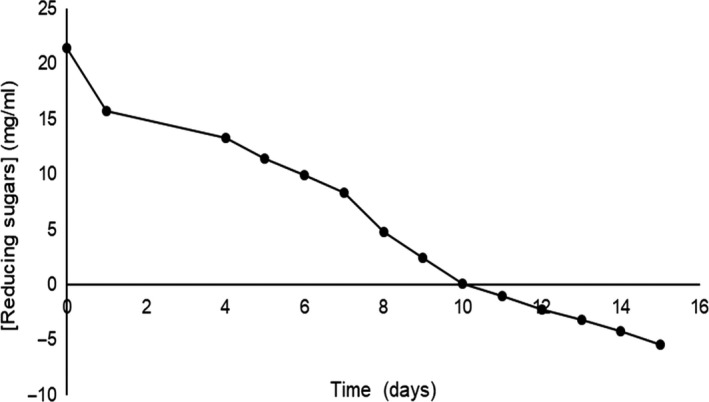
Glucose consumption in the production of bacterial cellulose by *Komagataeibacter xylinus*. The initial concentration of glucose in the media was 20 g/L

The determination of reducing sugars was performed based on the dinitrosalicylic acid (DNS) method. This analysis established a progressive decrease in the glucose content in the culture media, which is correlated with microbial growth. Figure 2 shows that the initial glucose concentration in the HS media was 20 g/L, and then in day 11, the concentration of reducing sugars was 21.46 mg/ml of glucose. Thus, the bacteria consumed the 99.44% (21.34 mg/ml) of the total consumed glucose. Furthermore, half of glucose in the media (10.08 mg/ml) was consumed the first 5 days of culture (Figure 2). Hestrin and Schramm (1954) mentioned that the optimum fermentation period for bacterial cellulose formation is 15 days. However, the reducing sugar analysis established that the optimal conditions for bacterial cellulose production in this study are 10 days. This reduction in time of bacterial cellulose production could be explained for the commercial strain of the bacteria used in this study. Thus, this commercial strain could be working much more effectively in the consumption of glucose and therefore production of bacterial cellulose.

#### 3.1.2 Scanning electron microscopy (SEM) of bacterial cellulose

**Figure 3:**
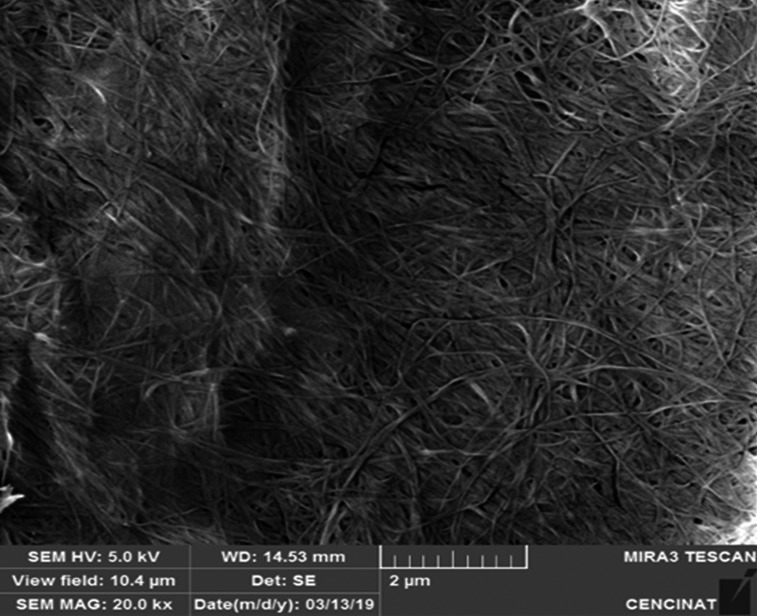
Morphology of bacterial cellulose produced by *Komagataeibacter xylinus* in HS media

The morphological image of bacterial cellulose obtained at the SEM analysis shows that the cellulose microfibrils have a single structure composed for ultrafine fibers forming the bacterial cellulose network (Semjonovs et al., 2017). Figure 3 also shows that the bacterial cellulose obtained in this study is ultrapure, with a higher crystallinity and degree of polymerization compared with the reported cellulose from plants (Römling & Galperin, 2015). Klemm, Schumann, Udhardt, and Marsch (2001) mention that the brown color and texture that are characteristic of the biofilm from the culture medium disappear with NaOH treatment, since the cellular and environmental residues are eliminated, producing crystallinity in the biofilm.

### 3.2 Physicochemical analysis of Castile blackberry coating

#### 3.2.1 Ultrafreezing storage of blackberry (−80°C)

##### Determination of titratable acidity of the blackberry (TA)

This analysis was performed every 5 days per treatment for 18 days. The titratable acidity decreased over time, showing a reduction in acidity from day 0 of 2.34% to 1.83% in day 18 for coated samples and 1.68% for uncoated samples.

The blackberry with coating is within the maximum pH for consumption (1.8%), according to the standard (NTE INEN 2427, 2010). While in the uncoated blackberry samples, the pH is out of the acceptable range of consumption according to the same standard. According to Wills and Burgos González (1998), the storage process of fruit at ultrafrozen temperatures causes loss of citric acid due to cryoscopic descent phenomena. In addition, Soares, Pires, and Azevedo (2014) demonstrated a decrease in citric acid during the first days in the storage of frozen pineapple. Thus, the reduction in citric acid of blackberries analyzed is consequence of the cryoscopic phenomena.

##### Determination of total soluble solids (°Brix) of the blackberry

An average value of 10.4°Brix in the sample with coating and 9.88°Brix for sample without coating was obtained. According to the standard (NTE INEN 2427, 2010), the total soluble solids of Castile blackberry must have a minimum of 9.0°Brix. The coated sample has concentrated sugars due to the cryoscopic phenomena mentioned previously, where there is a decrease in water and phenolic compounds and therefore increasing the °Brix. This shows one of the advantages in the implementation of bacterial cellulose coating, because the blackberry with coating has the highest capacity of retention of sugars contained in the fruit. Also, this organoleptic is an important characteristic for market purposes, due to most of the consumers prefer fruit with good level of sweetness in the different forms of consumption.

##### Maturity index after ultrafreezing storage

According to the standard (NTE INEN 2427, 2010), the limit of consumption of the blackberry is the maturity level 5, which is the minimum value. After 18 days, this index shows that the sample with coating is 6.1721, while the sample uncoated is 5.4054. This result is directly related to the cryoscopic phenomena, due to this index is calculated with the average values of acidity/°Brix (Wills & Burgos González, 1998).

In both treatments, the blackberry is in acceptable range of consumption. However, an interesting result is the increment of the useful life of the Castile blackberry with coating despite the maturity index of 6. Because the microbial load in the blackberry stored at −80°C was imperceptible even that the maturity index increased. This preservation does not happen with blackberries at room temperature condition, where the maturity index and the microbial load increase hand by hand.

##### Blackberry weight reduction

**Figure 4:**
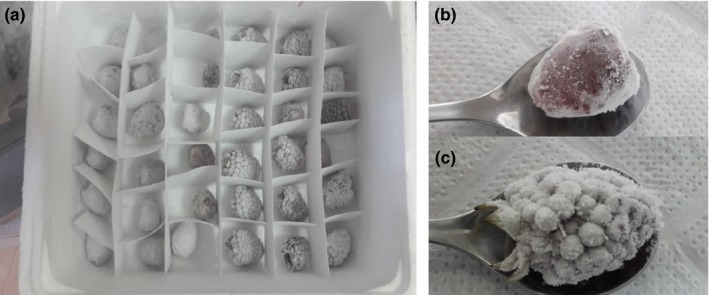
Weight loss of blackberry by ultrafreezing. (a) Tray used for the ultrafreezing experiment, (b) blackberry with coating, (c) and blackberry without coating

The ultrafreezing storage shows an apparent weight lost in the treatment with coating compared with the coated treatment. This result could be a response to the film acting as protector barrier for the sample, preventing the formation of micro crystals (nucleation phenomenon) that are part of the frost process in the fruit surface (Figure 4b,c). Therefore, it was possible to observe that the blackberries with coating did not gain humidity from the environment, leading to a lower weight than the initial one, having an average weight reduction of 2.05% after 18 days of ultrafreezing (Figure 4b). This result confirms that the bacterial cellulose acts as barrier to the blackberries when these were taken out from the freezer and no frost was observed in the sample (Figure 4b).

However, the uncoated samples showed less weight lost due to the phenomenon of volume change in the ultrafreezing since when taking out the sample from the freezer, it does not have a coating film that protects the blackberry, leading to the formation of the three‐dimensional structure of the hydrogen bridges. Therefore, the matrix is more hydrophilic tending to gain moisture, reflected in an average weight loss of 0.59% after 18 days of ultrafreezing (Figure 4c). Martín (2015) suggests that samples indirectly gain weight when its water content freezes, and the concentration of dissolved elements gradually increases. This phenomenon can be observed in blackberry when having no coating.

It is important to emphasize that in terms of organoleptic properties, better results were obtained in the treatment with bacterial cellulose. Since the color remained stable, unlike the uncoated sample had reduction in color after 18 days of deep freezing. Kopjar, Jakšić, and Piližota (2012) remark that addition of preservatives can prevent the decrease of anthocyanin content in stored blackberry. Thus, the biofilm acted as a protective barrier for the organoleptic properties in the fruit during the storage. This could be a reference methodology that can be used as alternative for exportation purpose of Blackberries. According to Mcguire (1992), color is a quality criteria considered by the consumers when selecting a product for its consumption. Color is also an important quality when determining the effectiveness of a postharvest treatment in fruits, being an important factor for its commercialization. Thus, an important factor in commercialization is the shelf life of the product.

#### 3.2.2 Shelf life analysis of the blackberry

The shelf life analysis was carried out at temperatures of 4°C ± 2; 25°C ± 2, and 37°C ± 2. The best treatment was at 4°C with coating, since the shelf life of 9 days was achieved, since it maintained its sensory properties compared with uncoated blackberry. After the day 9, the blackberry in this condition began to suffer physiological disorders such as dehydration, wrinkling, and aging. whereas the blackberry without coating just achieved a shelf life of 6 days at 4°C.

Also, at temperatures of 25°C ± 2 and 37°C ± 2, the blackberries with coating showed deterioration in the fifth and fourth day, respectively, while the uncovered berries showed deterioration around the third and second day, respectively. It is important to mention that at day 9, the blackberries in these conditions had dehydration, loss of color, very smooth texture, damage to its bark, and presence of mold.

In the bacterial analysis, there was no significant difference between treatment in the analysis with PDA agar, because the number of colonies obtained was low in relation to the growth of fungi and yeasts in both treatments in all temperatures (Table 1). While in the analysis with agar nutrient at temperatures of 4 and 37°C, no difference was showed since these two experiments are latent and accelerated, respectively. While at 25°C, a significant difference was obtained between treatments, showing that at this temperature the bacterial cellulose is effective in the conservation of the fruit (Table 2).

**Table 1:** Shelf life analysis of blackberry with and without coating on PDA agar

Temperature (°C)	Blackberry with coating (days)	Blackberry without coating (days)
4	9	9
25	4	4
37	3	3

**Table 2:** Shelf life analysis of blackberry with and without coating on nutrient agar

Temperature (°C)	Blackberry with coating (days)	Blackberry without coating (days)
4	17	14
25	14	7
37	7	6

## 4 CONCLUSIONS

The shelf life of Castile blackberry (*Rubus glaucus* Benth) with bacterial cellulose coating was up to 9 days at 4°C, giving a beneficial effect on the inhibition of microorganisms during the postharvest conservation of the fruit and providing physical and biological protection to the fruit. In addition, the sensory quality of the fruit at −80°C storage was not affected by the application of this coating, since the blackberries with coating had lower loss of color, compared with blackberries without coating, and positively influences the appearance of the fruit. Physicochemical properties such as titratable acidity, total soluble solids, maturity index, and weight reduction by ultrafreezing were stable during the 18 days of storage time, obtaining acceptable values within the norms established for their corresponding acceptability in the market.

## CONFLICT OF INTEREST

The authors declare no conflict of interest.

## ETHICAL APPROVAL

This study does not involve any human or animal testing.
