# CORRIGENDUM

**DOI:** 10.1002/fsn3.2305

**Published:** 2021-05-13

In the article by Toscano Ávila et al., (2020), there is an error in the second paragraph of the section 2.1. The correction is given below (in bold).

The acetic acid bacteria (BAA) Komagataeibacter xylinus strain **DSM2004** was used to produce the bacterial cellulose. Komagataeibacter xylinus strain was acquired from the Deutsche Sammlung von Mikroorganismen und Zellkulturen GmbH and collected, and it was provided by Dr. Pastor's group from the Department of Genetics, Microbiology and Statistics, Faculty of Biology, Universitat de Barcelona.
